# Double-stemmed and split structural variants of fluorescent RNA Mango aptamers

**DOI:** 10.1261/rna.079651.123

**Published:** 2023-09

**Authors:** Jeremy Herrera-Gutierrez, Steven J. Burden, Sarah E. Kobernat, Nicholas H. Shults, Mark Smith, Daniel Fologea, Eric J. Hayden

**Affiliations:** 1 Biomolecular Sciences Graduate Programs, Boise State University, Boise, Idaho 83725, USA; 2 Department of Biological Sciences, Boise State University, Boise, Idaho 83725, USA; 3 Department of Physics, Boise State University, Boise, Idaho 83725, USA

**Keywords:** fluorescent aptamer, RNA synthetic biology, RNA–RNA interaction, RNA nanotechnology

## Abstract

Aptamers with fluorogenic ligands are emerging as useful tools to quantify and track RNA molecules. The RNA Mango family of aptamers have a useful combination of tight ligand binding, bright fluorescence, and small size. However, the simple structure of these aptamers, with a single base-paired stem capped by a G-quadruplex, can limit the sequence and structural modifications needed for many use-inspired designs. Here we report new structural variants of RNA Mango that have two base-paired stems attached to the quadruplex. Fluorescence saturation analysis of one of the double-stemmed constructs showed a maximum fluorescence that is ∼75% brighter than the original single-stemmed Mango I. A small number of mutations to nucleotides in the tetraloop-like linker of the second stem were subsequently analyzed. The effect of these mutations on the affinity and fluorescence suggested that the nucleobases of the second linker do not directly interact with the fluorogenic ligand (TO1-biotin), but may instead induce higher fluorescence by indirectly altering the ligand properties in the bound state. The effects of the mutations in this second tetraloop-like linker indicate the potential of this second stem for rational design and reselection experiments. Additionally, we demonstrated that a bimolecular mango designed by splitting the double-stemmed Mango can function when two RNA molecules are cotranscribed from different DNA templates in a single in vitro transcription. This bimolecular Mango has potential application in detecting RNA–RNA interactions. Together, these constructs expand the designability of the Mango aptamers to facilitate future applications of RNA imaging.

## INTRODUCTION

Studying the dynamics and interactions of RNA molecules is central to understanding cellular regulatory processes. Recently, fluorogenic RNA aptamers have emerged as useful tools to detect and report intracellular RNA events due to their small size and genomic encodability (Dolgosheina et al. 2014; Pothoulakis et al. 2014; Ouellet 2016; Bouhedda et al. 2017; Braselmann et al. 2018; Yerramilli and Kim 2018). Typically, these “light-up aptamers” bind and enhance the fluorescence of a specific fluorogenic small molecule. Unlike fluorescent proteins, fluorogenic aptamers have not been found in nature, but instead have been developed in the laboratory using fluorescence-based or affinity-based in vitro selection (Ellington and Szostak 1990; Tuerk and Gold 1990; Bock et al. 1992). Research efforts are ongoing to improve both the binding and fluorescent response of these fluorogenic aptamer systems for better functionality in vivo and in vitro. This optimization requires sequence alterations that alter the structures of the RNA aptamers.

The RNA Mango family of aptamers has the promising combination of specific and tight ligand binding and significant fluorescence enhancement. The RNA Mango aptamers bind biotinylated derivatives of Thiazole Orange with low nanomolar *K*_*D*_ and have fluorescence enhancement of ∼1000-fold (Dolgosheina et al. 2014). There are currently several variants of RNA Mango with sequence variations that lead to different ligand affinities and fluorescence enhancements (Trachman et al. 2017; Autour et al. 2018; Kong et al. 2021). The structure of the Mango aptamers are all somewhat simple with a single base-paired stem capped by a G-quadruplex where the ligand binds. This simple structure is an advantage for efforts to integrate RNA Mango variants into RNA molecules of interest without disrupting structure or function. However, this structural simplicity is also limiting to the development of new functionalities. Other fluorogenic aptamers, such as RNA Spinach, also have G-quadruplex ligand binding sites, but with more than one base-paired stem joined to the quadruplex (Warner et al. 2014, 2017). These additional helical elements have been used in several applications that require more complex structures, including the detection of RNA–RNA interactions and dual-aptamer allosteric RNA biosensors (Paige et al. 2012; Rogers et al. 2015; Chandler et al. 2018). In addition, quadruplex flanking helices have been shown to modulate aptamer properties such as metal ion and ligand specificity (Ageely et al. 2016). While computational tools to predict quadruplex structures are advancing, they currently are not sufficient to predict how adding additional helical elements to a Mango quadruplex will alter affinity or fluorescence enhancement of the ligand.

**FIGURE 1.**
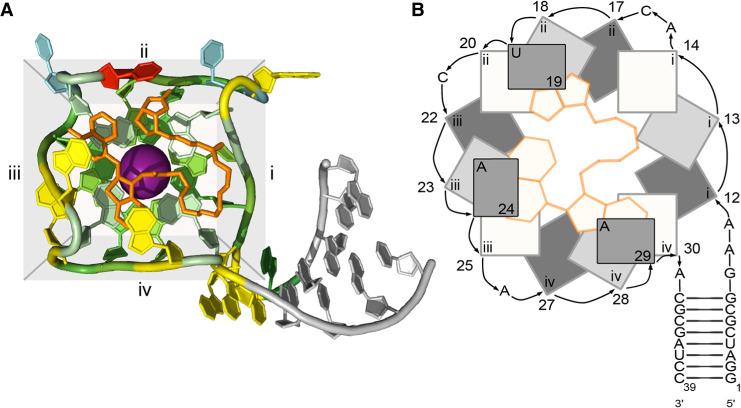
Structure of the Mango I aptamer. (*A*) Crystal structure of the aptamer bound to TO1-b (orange). The quadruplex is colored by residue, with G green, A yellow, C blue, and U red. The stem nucleotides are gray. Edges (i, ii, iii, and iv) are highlighted with a gray background. Structurally required potassium ion is shown as a purple sphere. (*B*) Abstract representation of the Mango I structure from the same perspective. Guanosine residues and A/U flaps are represented as rectangles. The edge that each guanosine belongs to is indicated by the Roman numeral inside the rectangle. Numbers represent nucleotide position 5′–3′. Covalent connectivity between residues is indicated by arrows. For guanosine residues, darker shading represents a lower position in the quadruplex tier. TO-1 biotin is shown as yellow.

Here we set out to design and characterize RNA Mango variants that have two base-paired stems attached to the G-quadruplex, which we will refer to as double-stemmed Mango (*ds*Mango). To facilitate the design process we developed an abstract representation based on the known structure of Mango I (Fig. 1; Trachman et al. 2017). Looking at this structure with the ligand-binding face of the G-quadruplex toward you, the structure appears square-like (Fig. 1A). Each edge of the square is comprised of three G residues that each contribute to one tier of the quadruplex. The convention in the literature is to name the four edges with Roman numerals (i, ii, iii, and iv) (Trachman et al. 2017). We will therefore refer to corners by a pair of Roman numerals. For example, the original Mango I structure shown in Figure 1A has the stem connected at corner i-iv, with the order of the Roman numerals indicating the order of the connected faces in the 5′–3′ direction. In the Mango I structure there are nucleotides within corners i-ii, ii-iii, and iii-iv, which are termed *loops*. The Mango I aptamer also requires that the stem is connected to the i-iv corner of the quadruplex with a 4 nt bulge region that is referred to as a *tetraloop-like motif*.

It has been shown previously that the Mango I aptamer can maintain fluorescence and ligand affinity when the stem is relocated to any of the corners of the quadruplex (Trachman et al. 2017). With this previously reported stem permutation experiment, combined with the multihelical structure of other fluorogenic aptamers, we reasoned that it should be possible to design double-stemmed variants of Mango I that maintain fluorescence and provide more design possibilities. The binding of TO1-3PEG-biotin (TO1-b) to the RNA Mango I aptamer includes interactions with nucleobases from the loops of the quadruplex. Specifically, the heterocyclic ring of TO1-b is stacked above by two adenosine residues and the biotin moiety interacts with the uracil through stacking and hydrogen bonds. These stacking nucleotides, termed “A/U flaps,” were highly conserved in molecules originally selected to bind TO1-b (Dolgosheina et al. 2014). This is important because any double-stemmed designs must not only enable proper quadruplex formation but must also allow these nucleotide flaps to interact favorably with the ligand.

## RESULTS AND DISCUSSION

### Design of double-stem sequences

**FIGURE 2.**
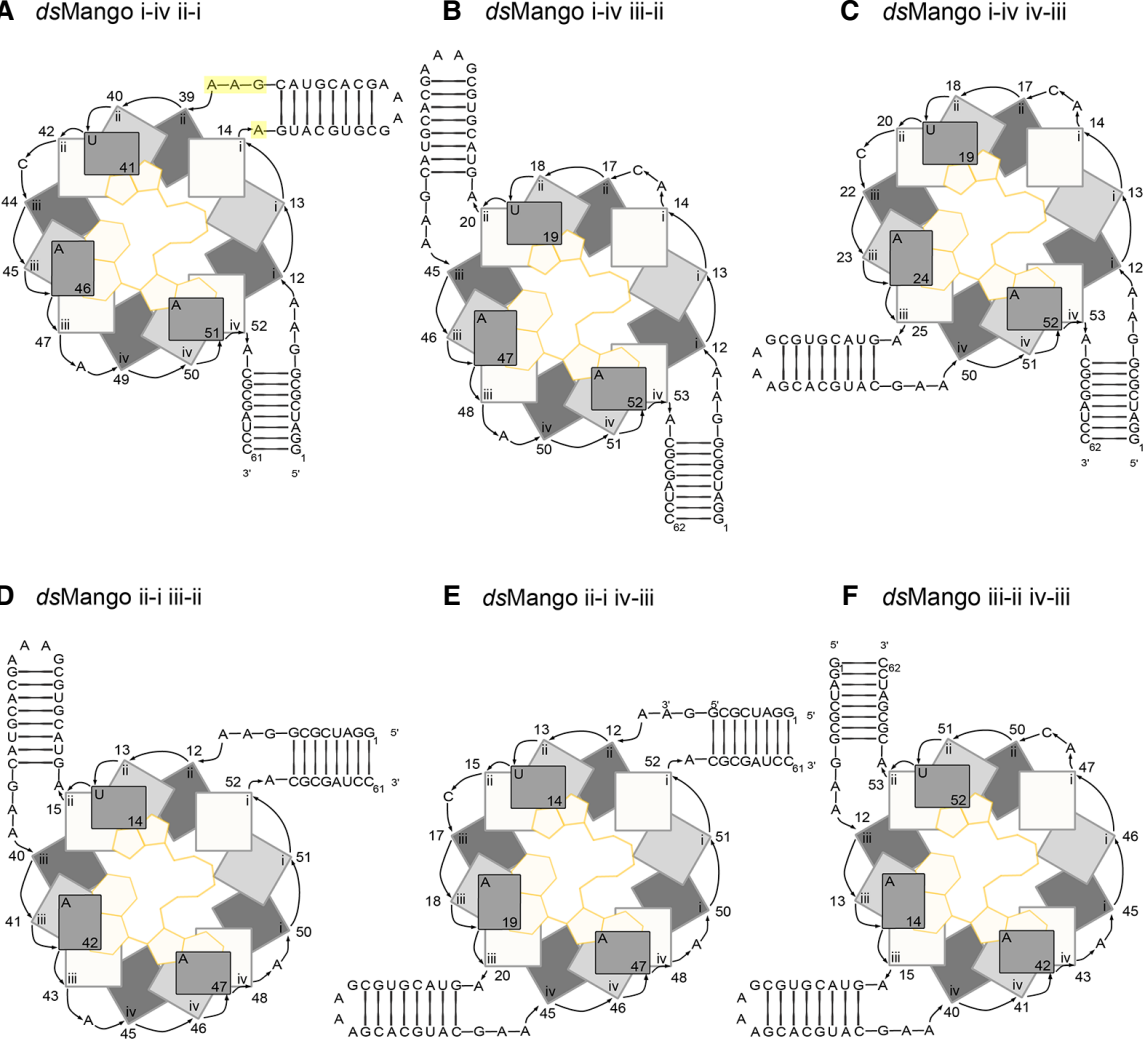
Abstract representations of the *ds*Mango variants. (*A*) *ds*Mango i-iv ii-i with the first stem in the i-iv position and second stem attachment point at the ii-i corner. Nucleotides of the tetraloop-like motif is highlighted in yellow. These nucleotides were mutated in subsequent experiments. (*B*) *ds*Mango i-iv iii-ii with the first stem in the i-iv position and second stem attachment point at the iii-ii corner. (*C*) *ds*Mango i-iv iv-iii with the first stem in the i-iv position and second stem attachment point at the iv-iii corner. (*D*) *ds*Mango ii-i iii-ii with the first stem in the ii-i position and second stem attachment point at the iii-ii corner. (*E*) *ds*Mango ii-i iv-iii with the first stem in the ii-i position and second stem attachment point at the iv-iii corner. (*F*) *ds*Mango iii-ii iv-iii with the first stem in the iii-ii position and second stem attachment point at the iv-iii corner.

To facilitate proper folding of *ds*Mango constructs, we first set out to design two different stems with unique base compositions. For one stem, the originally reported 8 bp stem of RNA Mango I was used. The sequence of the second stem was chosen from several designs because it was predicted to form the designed base pairs in both stems with very high probability and with very low probability of other base pair interactions based on thermodynamic structure prediction (Nupack) (Supplemental Fig. S1). Both stems had GAAA tetraloop-like linkers where the stems connected to the G-quadruplex (Fig. 2, highlighted). Next, six unique *ds*Mango constructs were designed by joining the two stems to different corners of the core G-quadruplex (Fig. 2). These designs were named by the corners where each stem was joined to the quadruplex. For example, *ds*Mango i-iv ii-i has the first stem connected at the i-iv corner and the second stem in the ii-i corner. The first stem in the name is always formed by the 5′ and 3′ ends of the molecule, and the second stem in the name is capped by a GAAA tetraloop. Importantly, each stem replaces at least 1 nt in the loops when inserted into the G-quadruplex. This was identical to the previously published permutation experiment, which confirmed ligand binding and fluorescence for each single-stem location (Trachman et al. 2017). Also based on this previous report, we added a single adenosine residue to corner i-iv in designs that did not have a stem at this corner to allow for the quadruplex formation.

### Determining maximum fluorescence and ligand affinity for *ds*Mango variants

**FIGURE 3.**
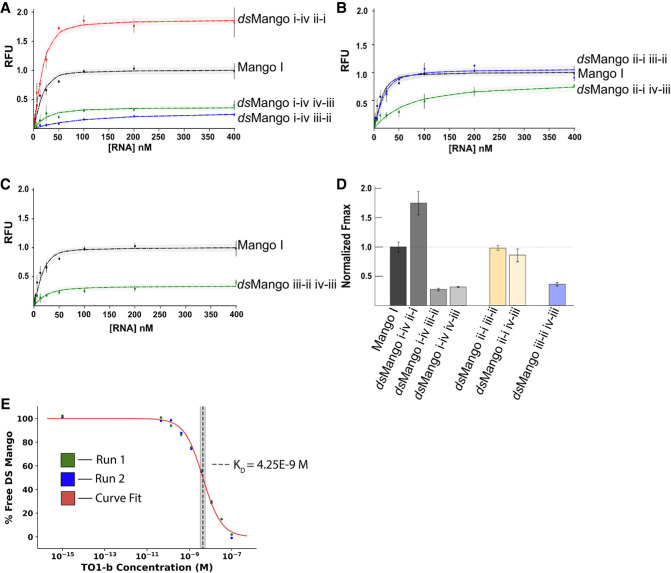
Characterization of the *ds*Mango variants. (*A*–*C*) Fluorescence saturation curves with 25 nM TO1-b and variable RNA concentrations. Shaded regions show 68% CI of the curve fit. Constructs are grouped based on whether the first stem is at (*A*) the i-iv corner, (*B*) the ii-i corner, or (*C*) the iii-ii corner. (*D*) Relative maximum fluorescence (*F*_max_) of the double-stemmed variants. (*E*) Fraction of *ds*Mango i-iv ii-i in free solution over a range of TO1-b concentrations. Curve fit line represents least squares fit using Equation 1 (see Materials and Methods). *K*_*D*_ derived from curve fitting is indicated by the dotted line. The shaded region representing the 95% CI is determined from error curve analysis.

To evaluate the fluorescence and ligand affinity of the six *ds*Mango variants, the fluorescence was measured at a range of concentrations of RNA, with a fixed TO1-b concentration. Nonlinear curve fitting was used to determine *F*_max_ and *K*_*D*_ (see Materials and Methods). *F*_max_ values for *ds*Mango variants are reported as relative to the fluorescence observed for the single-stemmed Mango I (Fig. 3). Not surprisingly, four variants had very low ligand affinity and poor fluorescence enhancement (Table 1; Fig. 3A–C). However, the *ds*Mango i-iv ii-i and *ds*Mango ii-i iii-ii variants had maximum fluorescence values as bright or brighter than the single-stemmed Mango I. In addition, the two variants had *K*_*D*_ values in the low nanomolar range (*K*_*D*_ = 4.66 ± 2.7, 6.32 ± 1.2), very similar to single-stemmed Mango I. The brightest double-stemmed construct was *ds*Mango i-iv ii-i, which was ∼75% brighter than the single-stem Mango I (*F*_max_ = 1.75 ± 0.08). In addition to curve fitting, the *K*_*D*_ for *ds*Mango i-iv ii-i was validated using a KinExA binding affinity assay (see Materials and Methods). This technique measures the unbound Mango aptamer remaining on an affinity column after mixing with a series of TO1-b dilutions. The resulting data was fit using KinExA software which resulted in a measured *K*_*D*_ of 4.35 nM (95% CI of 3.28–5.57 nM) (Fig. 3E). Of note, the *ds*Mango i-iv ii-i construct and the original Mango I share the same first stem connection point at corner i-iv, but the second brightest double-stemmed construct *ds*Mango ii-i iii-ii used a different corner for the first stem and still showed a fluorescence saturation curve nearly identical to Mango I (*F*_max_ = 0.98 ± 0.04). This demonstrates that the i-iv corner is not a requirement for double-stemmed designs (Fig. 3B). The third brightest construct *ds*Mango ii-i iv-iii, was not quite as bright as the original single-stemmed Mango I (*F*_max_ = 0.86 ± 0.11), and showed reduced ligand affinity (Fig. 3B, green curve).

**TABLE 1.**
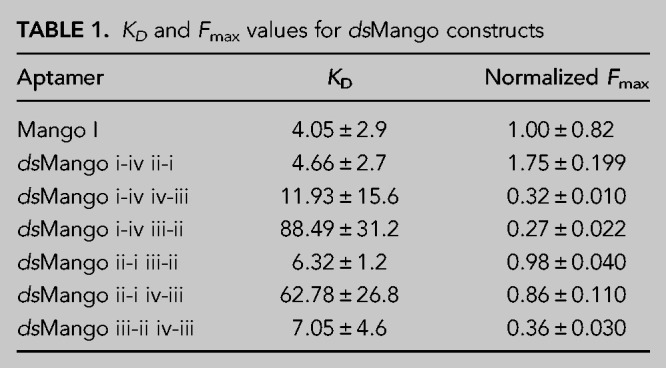
*K*_*D*_ and *F*_max_ values for *ds*Mango constructs

### Possible mechanisms of increased *F*_max_

We considered several ways that the *ds*Mango i-iv ii-i construct could achieve brighter maximum fluorescence than the single-stemmed Mango I. The presence of a second stem adds thermodynamic stability from additional base pairs, which could result in a greater fraction of correctly folded RNA molecules. However, because the concentration of RNA is saturating, this mechanism in isolation would require an ∼75% increase in the fraction folded. Alternatively, the second stem could create a new tertiary interaction that alters the quadruplex core. This has been previously observed in the structure of Mango III, where a pseudoknot-like tertiary interaction leads to structural rearrangements at the ligand binding site that alters the planarity of the TO1 in the bound state leading to an increased fluorescence enhancement relative to Mango I (Trachman et al. 2019). However, it is not obvious how such a tertiary structure could form in the two-stemmed construct given the noninteracting nature of the single stem in the crystal structure of Mango I. A third possibility is that the nucleotides of the second stem of *ds*Mango i-iv iii-ii interact directly with the TO1-B ligand or the A/U flaps, altering fluorescence enhancement. For example, because these nucleobases of the tetraloop-like linker in the second stem are not involved in canonical base pairs and may be in close proximity to the fluorophore and A/U flaps, it is possible that the nucleobases in the second tetraloop-like linker could directly interact with the ligand through hydrogen bonding or stacking. Alternatively, the second stem could indirectly cause a subtle change to the final bound state to increase fluorescence without the linker nucleobases directly interacting with the ligand.

### Effects of mutations to the ii-i tetraloop-like linker

To better understand whether or not the nucleotides of the tetraloop-like linker contribute to the fluorescence enhancement caused by the second stem, we measured the fluorescence of a series of sequences with mutations in the second tetraloop-like linker. Fluorescence saturation curves were examined for nine mutants and nonlinear curve fitting was used to determine *K*_*D*_ and *F*_max_ (Table 2; Supplemental Figs. S3, S4). *F*_max_ was measured as relative to the unmutated *ds*Mango i-iv ii-i, which was remeasured side by side with the mutational variants as a control. Most of the individual mutations had very little effect on *F*_max_ or *K*_*D*_. We conclude that it is unlikely that nucleobases in the linker make direct hydrogen bonds or stacking interactions with the TO1-b ligand. Interestingly, some of the individual mutations appear to have slightly lower *K*_*D*_ values in these experiments. We are hesitant to make strong conclusions about this enhanced binding affinity because the curve fitting in this experimental design is sensitive to experimental noise. In addition, some of the sequence variants appeared to have decreased binding affinity, including some individual mutations, a double mutant, and a variant where the GAAA tetraloop was completely replaced with a different tetraloop CUUG. It is likely that the decreased affinity in these cases was caused by misfolding events. Nevertheless, the results indicate the potential to modify ligand affinity by altering the linker sequence. These results suggest that further exploration of sequence variants may be warranted, which could be accomplished through randomizations, expansions, and reselections (Trachman et al. 2019; Dey et al. 2022).

**TABLE 2.**
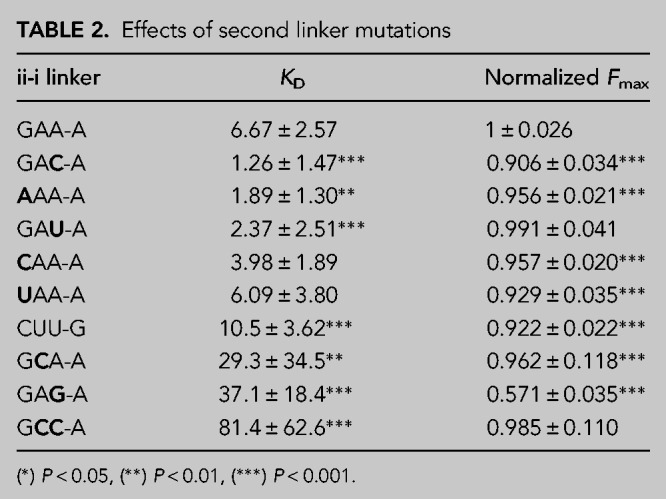
Effects of second linker mutations

### Demonstration of split RNA Mango

**FIGURE 4.**
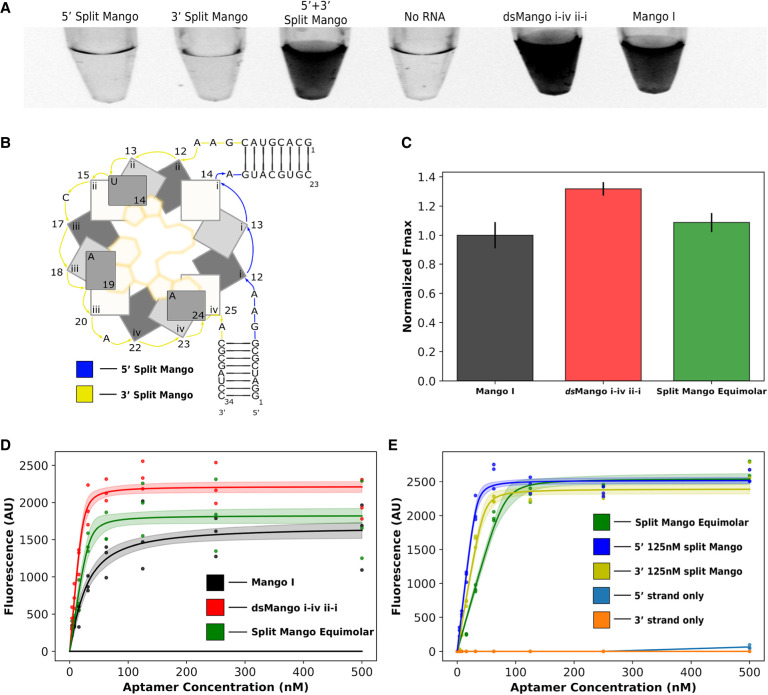
Fluorescence of the split *ds*Mango i-iv ii-i (split Mango) construct. (*A*) Image of fluorescent response for 5′ split Mango strand only, 3′ split Mango strand only, both strands of split Mango, *ds*Mango i-iv ii-i, and Mango I. Color is inverted such that fluorescence appears dark. (*B*) Abstract representation of split Mango with the 5′ strand (blue) and 3′ strand (yellow) highlighted. (*C*) Comparison of *F*_max_ for split Mango (equimolar 5′,3′) and *ds*Mango i-iv ii-i normalized to Mango I. (*D*) Fluorescence saturation curves comparing response of Mango I, *ds*Mango i-iv ii-i, and split Mango (equimolar 5′,3′). (*E*) Fluorescence saturation curves for split Mango where either the 5′ or 3′ are held at 125 nM while the complementary strand is varied. Fluorescence of the split Mango 5′ and 3′ strand alone was also evaluated. Shaded regions and error bars represent 68% CI.

We anticipated that the *ds*Mango could remain functional when split into two separate strands. To design this bimolecular construct, the tetraloop capping the second stem was removed to split the structure into two separate RNA sequences (Fig. 4B). Split *ds*Mango i-iv ii-i (“split Mango”) was first investigated by separately transcribing and PAGE purifying each strand of the split sequence, and then mixing them back together in 1× Mango buffer with TO1-b (Fig. 4). A simple visual appraisal in tubes confirmed that split Mango achieved fluorescence comparable to the unimolecular *ds*Mango (Fig. 4A). The individual single strands alone showed fluorescence levels indistinguishable from the background fluorescence of TO1-b (“No RNA” control). To further characterize this split construct, fluorescence was measured across a range of RNA concentrations with both strands at equimolar concentrations, and this fluorescence was compared to unimolecular *ds*Mango (Fig. 4C,D). The split designs showed an apparent *F*_max_ that was slightly lower than unimolecular *ds*Mango i-iv ii-i, and had a higher apparent *K*_*D*_, requiring slightly more RNA to achieve saturation (Fig. 4D). This suggests that the split construct might have some folding inefficiencies, possibly caused by structure within either of the individual stands. To further explore this idea, the fluorescent response of split Mango was evaluated when one of the two strands was kept at a fixed concentration of 125 nM while the second strand was varied (0–500 nM) (Fig. 4E). In both cases, the apparent *K*_*D*_ improved slightly while the *F*_max_ was not affected (Table 3). The results suggest that neither of the individual strands has a strongly interfering intramolecular structure, and that equimolar concentrations of both strands is not necessary and may not be optimal for bimolecular folding. This is promising for intracellular applications where the concentration of each strand may not be identical.

**TABLE 3.**
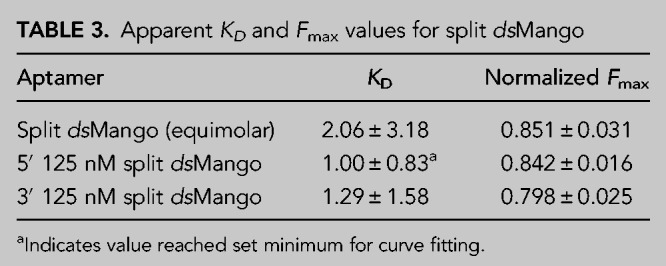
Apparent *K*_*D*_ and *F*_max_ values for split *ds*Mango

### Cotranscriptional fluorescence

**FIGURE 5.**
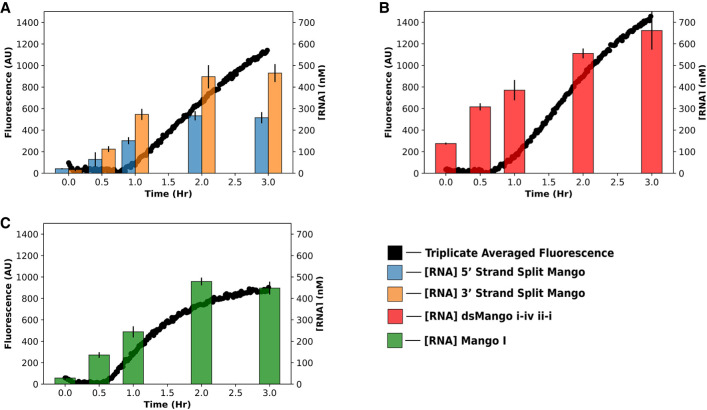
Cotranscriptional fluorescence of unimolecular and split Mango constructs. Fluorescence (*left, y*-axis) and RNA concentration (*right, y*-axis) over the course of transcription time (*x*-axis) for (*A*) unimolecular *ds*Mango i-iv ii-i, (*B*) unimolecular Mango I, and (*C*) split Mango. Fluorescence data represents the mean at that time (*n* = 3). Error bars on RNA concentration represent CI of 68%. Fluorescence and RNA were separate reactions under identical conditions.

To further evaluate double-stemmed and split Mango functionality under more biologically relevant conditions, the fluorescence signal over time was measured during an in vitro transcription reaction containing Mango buffer, TO1-b, and two separate DNA templates. Change in RNA concentration was also measured during a separate transcriptional time-course, under the same conditions, using band quantification after denaturing PAGE. The results showed an increase in fluorescence over time under these cotranscriptional conditions (Fig. 5A, black data points). The relative fluorescence signal over time for the split construct was similar to the unimolecular *ds*Mango, indicating efficient bimolecular complex formation (Fig. 5B, black data points). All constructs, including the unimolecular Mango I (Fig. 5C), showed a slight lag from when RNA was easily detectable and when fluorescence increased. This is possibly caused by inefficient heating in the plate reader where fluorescence was measured, as compared to the thermocycler where RNA was transcribed for gel analysis. However, in a previous publication, NMR studies showed an induced fit mechanism for ligand binding for Mango III, which could be contributing to the lag time between RNA transcription and fluorescence in our cotranscriptional reactions (Harish et al. 2022). Nevertheless, the results clearly indicate that the split construct can fold and fluoresce when transcribed from separate DNA templates, which is promising for future applications.

### Conclusions

The double-stemmed RNA Mango constructs reported here provide new design options for fluorogenic Mango aptamers. For example, the bright double-stemmed RNA mango aptamer opens up the possibilities for functionalization and use in biosensors (Su and Hammond 2020). While it remains difficult to predict unwanted sequence interactions that might occur during different applications, having two different double-stemmed constructs that do not compromise ligand affinity or maximum fluorescence provides options for challenging designs, such as those involving long range interactions. The increased maximum fluorescence of *ds*Mango i-iv ii-i relative to Mango I might prove useful for certain applications, and creates interesting structural questions. The effects on both binding affinity and maximum fluorescence caused mutations in the second tetraloop-like linker further suggest that the double-stem Mango construct has new structural interactions with the TO1-b ligand that are different than the Mango I. Further investigations are needed to identify direct and indirect interactions between the second stem and the ligand. The changes in fluorescence and affinity with small sequence changes highlights the challenges that remain for RNA structural design and fluorescent aptamer engineering, especially when noncanonical structures such as G-quadruplexes are important for function.

The split RNA Mango aptamer could be useful for the study of RNA–RNA interactions. For example, each half of the aptamer could be placed on a different RNA molecule, and fluorescence could be used to detect, track, and quantify when the two RNA molecules interact (Alam et al. 2017; Kolpashchikov and Spelkov 2021). RNA–RNA interactions are critical for numerous processes including spliceosomal assembly and function, RNA modification, RNA interference pathways, bacterial small RNA, and CRISPR/Cas systems, among others. In addition, the simple split designs reported here could function as a rapid fluorescence-based assay for RNA helicase activity that could even be genetically encoded (Jarmoskaite and Russell 2014). Our split designs could also be useful in designing genetically encodable FRET systems and other RNA origami applications (Jepsen et al. 2018). Overall, these new *ds*Mango aptamers expand the design potential of RNA Mango and create new opportunities for studying RNA structure and dynamics.

## MATERIALS AND METHODS

### RNA transcription and purification

Mango variants were made by in vitro transcription from synthetic oligonucleotide templates (IDT). The T7 promoter regions of each template were made double-stranded by annealing a second oligonucleotide with the sequence of the top strand of the promoter. DNA was heated to 95°C for 5 min and cooled to room temperature in 1× T7 buffer (50 mM Tris pH 7.5, 20 mM MgCl_2_, 5 mM DTT, and 2 mM spermidine). This primer-annealed template was added to a transcription reaction containing 1× T7 buffer, 2.5 mM ribonucleotide triphosphate mix (NEB: N0466S), and ∼800 units of T7 RNA polymerase (Thermo Fisher Scientific: EP0113). RNA was purified on a 12% polyacrylamide gel with 8 M urea. Bands were visualized with a brief exposure to UV light, and excised with sterile razor blades. Gel was crushed and eluted in 300 mM sodium acetate (RNase Free) at room temperature for 1 h. The RNA eluted from the crushed gel was filtered by centrifugation through a 0.2 µm sterile filter and ethanol precipitated with 3 volumes of 95% cold ethanol at −20°C for 5 min. RNA was pelleted by centrifugation at 4°C for 20 min and washed with 80% then 95% ethanol. Pellets were dried in a SpeedVac for ∼2 min, then rehydrated in RNase free water and quantified by A260 (NanoDrop). Purified RNA was run again on an analytical 12% denaturing polyacrylamide gel for quality control.

### Binding affinity and maximum fluorescence

Serial dilutions of RNA were made using a 1× Mango buffer (10 mM NaH_2_PO_4_ pH 7.4, 1 mM MgCl_2_, and 140 mM KCl). Upon addition of TO1-3PEG-biotin (Applied Biological Materials), relative fluorescence was measured with a DeNovix QFX fluorometer. Best-fit curves were generated using the nonlinear regression function from the Scipy_Optimize package in python. The averages of the triplicate data were fit using a Trust Region Reflective least squares algorithm to Equation 1 (below) where [*apt*] and [*TO1b*] are the nanomolar concentrations of RNA and TO1-3PEG-biotin, respectively. *F*′ is the molar maximum fluorescence which is the *F*_max_/[*TO1b*]. The 68% confidence intervals were calculated using the Python uncertainties package.

(1) $$\begin{array}{rl} & F(\left[apt\right])=\\ & \frac{F^{\prime }\left[(K_{D}+[apt]+[TO1b])-\;\sqrt{\left((\left[apt]-{\left[TO1b]\right)}^{2}+K_{D}(K_{D}+2[apt]+2[TO1b]\right)\right)}\;\right]}{2} \end{array}$$

### Affinity and fluorescence of tetraloop-like linker mutations

The variants with mutations in the tetraloop-like linker were evaluated very similar to above, but with the following differences. PAGE purified RNA was prepared as described above. For each double-stemmed construct, twofold serial dilutions were prepared and individually loaded onto 96 well plates. Dilutions ranged from 800 nM to 6.25 nM except for the A38 series of mutants which ranged from 500 nM to 3.9 nM. For each reaction, 1× Mango buffer containing TO1-b at a concentration of 40 nM was added prior to being briefly spun down. The samples were then incubated for 2 min at 65°C and cooled to room temperature for 2 min prior to measurement on a Tecan Spark Plate reader at 510 nm excitation, 535 nm emission. Triplicate data values were fit using the nonlinear regression procedure described above. Statistically significant changes in *K*_*D*_ and *F*_max_ for each mutation were determined by Student's unpaired *t*-test (*n* = 20).

### Cotranscriptional fluorescence

Mango RNA was prepared cotranscriptionally in triplicate from 1 µM synthetic oligonucleotide templates and 1 µM T7 promoter in the presence of T7 buffer, ∼400 units of T7 RNA polymerase, 1× Mango buffer, and 40 nM TO1-b. Two sets of identical plates were prepared simultaneously and individually ran on a preheated Eppendorf MasterCycler and Tecan Spark plate reader at 37°C for 3 h. Fluorescence measurements (510 nm excitation, 545 nm emission) were taken continuously in 1 min intervals from the plate reader. RNA concentration values were determined from 40 µL samples taken from the MasterCycler run plate which were collected at 0, 0.5, 1, 2, and 3 h timepoints and stored at −20°C. Band separation of the sample products was performed using analytical 12% Urea-PAGE gels. Gels were stained in Gel Red for 30 min and subsequently imaged. Densitometry was performed using GelAnalyzer using morphological background subtraction (10% width tolerance). Density values for each sample were compared to a purified RNA concentration reference to estimate sample RNA concentrations.

### Split RNA/unimolecular Mango preparation and fluorescent measurement

Split RNA aptamers were transcribed and purified separately as described above. Mango I and *ds*Mango were also prepared similarly. From this RNA, ½ serial dilutions were performed to obtain the specified concentration. For each concentration sample, purified RNA aptamer strands were individually loaded onto 96 well plates with 1× Mango buffer containing TO1-b at a concentration of 40 nM. The plate was then briefly spun down before incubation at 70°C for 3 min. After incubation, the plate was cooled to room temperature for 5 min prior to fluorescent measurement on a Tecan Spark plate reader at 510 nm excitation, 535 nm emission. Triplicate data values were fit using the nonlinear regression procedure described above.

### KinExA fluorescent measurement assays

The dissociation constant was measured by an affinity exclusion assay with a KinExA model 3200 (Sapidyne). RNA (5 nM) in 1× Mango buffer was mixed with fivefold dilutions of TO1-3PEG-biotin beginning with 1.0 µM. Samples were also supplemented with 0.02% tween-20 to minimize nonspecific binding to the KinExA tubing system. Samples were allowed to equilibrate for 4 h at room temperature. The stationary phase of the system consisted of polymethylmethacrylate beads functionalized with TO1-3PEG-biotin according to Sapidyne protocols. Following introduction of the sample, buffer was flowed across the flow cell for 4 min at 0.25 mL/min. The amount of free aptamer from each equilibrium mixture was quantified by the fluorescent signal resulting from the binding of this free aptamer to the TO1-b functionalized beads in the stationary phase. *K*_*D*_ was calculated by the KinExA software using the equilibrium protocol.

## SUPPLEMENTAL MATERIAL

Supplemental material is available for this article.

## References

[RNA079651HERC1] Ageely EA, Kartje ZJ, Rohilla KJ, Barkau CL, Gagnon KT. 2016. Quadruplex-flanking stem structures modulate the stability and metal ion preferences of RNA mimics of GFP. ACS Chem Biol 11: 2398–2406. 10.1021/acschembio.6b00047 27467146

[RNA079651HERC2] Alam KK, Tawiah KD, Lichte MF, Porciani D, Burke DH. 2017. A fluorescent split aptamer for visualizing RNA–RNA assembly *in vivo*. ACS Synth Biol 6: 1710–1721. 10.1021/acssynbio.7b00059 28548488 PMC5603824

[RNA079651HERC3] Autour A, Jeng SCY, Cawte AD, Abdolahzadeh A, Galli A, Panchapakesan SSS, Rueda D, Ryckelynck M, Unrau PJ. 2018. Fluorogenic RNA Mango aptamers for imaging small non-coding RNAs in mammalian cells. Nat Commun 9: 656. 10.1038/s41467-018-02993-8 29440634 PMC5811451

[RNA079651HERC4] Bock LC, Griffin LC, Latham JA, Vermaas EH, Toole JJ. 1992. Selection of single-stranded DNA molecules that bind and inhibit human thrombin. Nature 355: 564–566. 10.1038/355564a0 1741036

[RNA079651HERC5] Bouhedda F, Autour A, Ryckelynck M. 2017. Light-up RNA aptamers and their cognate fluorogens: from their development to their applications. Int J Mol Sci 19: 44. 10.3390/ijms19010044 29295531 PMC5795994

[RNA079651HERC6] Braselmann E, Wierzba AJ, Polaski JT, Chromiński M, Holmes ZE, Hung S-T, Batan D, Wheeler JR, Parker R, Jimenez R, 2018. A multicolor riboswitch-based platform for imaging of RNA in live mammalian cells. Nat Chem Biol 14: 964–971. 10.1038/s41589-018-0103-7 30061719 PMC6143402

[RNA079651HERC7] Chandler M, Lyalina T, Halman J, Rackley L, Lee L, Dang D, Ke W, Sajja S, Woods S, Acharya S, 2018. Broccoli fluorets: split aptamers as a user-friendly fluorescent toolkit for dynamic RNA nanotechnology. Molecules 23: E3178. 10.3390/molecules23123178 PMC6321606 30513826

[RNA079651HERC8] Dey SK, Filonov GS, Olarerin-George AO, Jackson BT, Finley LWS, Jaffrey SR. 2022. Repurposing an adenine riboswitch into a fluorogenic imaging and sensing tag. Nat Chem Biol 18: 180–190. 10.1038/s41589-021-00925-0 34937909 PMC8967656

[RNA079651HERC9] Dolgosheina EV, Jeng SCY, Panchapakesan SSS, Cojocaru R, Chen PSK, Wilson PD, Hawkins N, Wiggins PA, Unrau PJ. 2014. RNA Mango aptamer-fluorophore: a bright, high-affinity complex for RNA labeling and tracking. ACS Chem Biol 9: 2412–2420. 10.1021/cb500499x 25101481

[RNA079651HERC10] Ellington AD, Szostak JW. 1990. *In vitro* selection of RNA molecules that bind specific ligands. Nature 346: 818–822. 10.1038/346818a0 1697402

[RNA079651HERC11] Harish B, Wang J, Hayden EJ, Grabe B, Hiller W, Winter R, Royer CA. 2022. Hidden intermediates in Mango III RNA aptamer folding revealed by pressure perturbation. Biophys J 121: 421–429. 10.1016/j.bpj.2021.12.037 34971617 PMC8822612

[RNA079651HERC12] Jarmoskaite I, Russell R. 2014. RNA helicase proteins as chaperones and remodelers. Annu Rev Biochem 83: 697–725. 10.1146/annurev-biochem-060713-035546 24635478 PMC4143424

[RNA079651HERC13] Jepsen MDE, Sparvath SM, Nielsen TB, Langvad AH, Grossi G, Gothelf KV, Andersen ES. 2018. Development of a genetically encodable FRET system using fluorescent RNA aptamers. Nat Commun 9: 18. 10.1038/s41467-017-02435-x 29295996 PMC5750238

[RNA079651HERC14] Kolpashchikov DM, Spelkov AA. 2021. Binary (split) light-up aptameric sensors. Angew Chem Int Ed Engl 60: 4988–4999. 10.1002/anie.201914919 32208549

[RNA079651HERC15] Kong KYS, Jeng SCY, Rayyan B, Unrau PJ. 2021. RNA Peach and Mango: orthogonal two-color fluorogenic aptamers distinguish nearly identical ligands. RNA 27: 604–615. 10.1261/rna.078493.120 33674421 PMC8051271

[RNA079651HERC16] Ouellet J. 2016. RNA fluorescence with light-up aptamers. Front Chem 4: 29. 10.3389/fchem.2016.00029 27446908 PMC4923196

[RNA079651HERC17] Paige JS, Nguyen-Duc T, Song W, Jaffrey SR. 2012. Fluorescence imaging of cellular metabolites with RNA. Science 335: 1194. 10.1126/science.1218298 22403384 PMC3303607

[RNA079651HERC18] Pothoulakis G, Ceroni F, Reeve B, Ellis T. 2014. The spinach RNA aptamer as a characterization tool for synthetic biology. ACS Synth Biol 3: 182–187. 10.1021/sb400089c 23991760

[RNA079651HERC19] Rogers TA, Andrews GE, Jaeger L, Grabow WW. 2015. Fluorescent monitoring of RNA assembly and processing using the split-spinach aptamer. ACS Synth Biol 4: 162–166. 10.1021/sb5000725 24932527

[RNA079651HERC20] Su Y, Hammond MC. 2020. RNA-based fluorescent biosensors for live cell imaging of small molecules and RNAs (biosensor review). Curr Opin Biotechnol 63: 157–166. 10.1016/j.copbio.2020.01.001 32086101 PMC7308196

[RNA079651HERC21] Trachman RJ, Demeshkina NA, Lau MWL, Panchapakesan SSS, Jeng SCY, Unrau PJ, Ferré-D'Amaré AR. 2017. Structural basis for high-affinity fluorophore binding and activation by RNA Mango. Nat Chem Biol 13: 807–813. 10.1038/nchembio.2392 28553947 PMC5550021

[RNA079651HERC22] Trachman RJ, Autour A, Jeng SCY, Abdolahzadeh A, Andreoni A, Cojocaru R, Garipov R, Dolgosheina EV, Knutson JR, Ryckelynck M, 2019. Structure and functional reselection of the Mango-III fluorogenic RNA aptamer. Nat Chem Biol 15: 472–479. 10.1038/s41589-019-0267-9 30992561 PMC7380332

[RNA079651HERC23] Tuerk C, Gold L. 1990. Systematic evolution of ligands by exponential enrichment: RNA ligands to bacteriophage T4 DNA polymerase. Science 249: 505–510. 10.1126/science.2200121 2200121

[RNA079651HERC24] Warner KD, Chen MC, Song W, Strack RL, Thorn A, Jaffrey SR, Ferré-D'Amaré AR. 2014. Structural basis for activity of highly efficient RNA mimics of green fluorescent protein. Nat Struct Mol Biol 21: 658–663. 10.1038/nsmb.2865 25026079 PMC4143336

[RNA079651HERC25] Warner KD, Sjekloća L, Song W, Filonov GS, Jaffrey SR, Ferré-D'Amaré AR. 2017. A homodimer interface without base pairs in an RNA mimic of red fluorescent protein. Nat Chem Biol 13: 1195–1201. 10.1038/nchembio.2475 28945234 PMC5663454

[RNA079651HERC26] Yerramilli VS, Kim KH. 2018. Labeling RNAs in live cells using malachite green aptamer scaffolds as fluorescent probes. ACS Synth Biol 7: 758–766. 10.1021/acssynbio.7b00237 29513000

